# Ionically crosslinked biohybrid gelatin-based hydrogels for 3D cell culture

**DOI:** 10.1007/s13233-025-00380-z

**Published:** 2025-02-19

**Authors:** Eric Y. Du, H. T. Kim Duong, M. A. Kristine Tolentino, Jacinta L. Houng, Panthipa Suwannakot, Kristel C. Tjandra, Duyen H. T. Nguyen, Richard D. Tilley, J. Justin Gooding

**Affiliations:** 1 School of Chemistry, The University of New South Wales, Sydney, NSW 2032 Australia; 2 Australian Centre for NanoMedicine, The University of New South Wales, Sydney, NSW 2032 Australia; 3 Electron Microscopy Unit, Mark Wainwright Analytical Centre, The University of New South Wales, Sydney, NSW 2032 Australia

**Keywords:** Hydrogel, ECM mimic, Polymer, Gelatin

## Abstract

The transition from two-dimensional to three-dimensional cell cultures has transformed the understanding of cell physiology and cell–matrix interactions. Extracellular matrix (ECM) mimics tend to fall into either the natural or synthetic categories. Naturally occurring ECM mimics, such as collagen and gelatin, have superior bioactive properties but typically lack tuneability. Conversely, synthetic ECM mimics are highly defined but even with modifications, can lack the bioactivity of natural proteins. Therefore, to take advantage of the potential of both natural and synthetic ECM mimics, a biohybrid ionically crosslinked gelatin hydrogel was synthesised. This was achieved by utilising free amine groups along the gelatin backbone as the basis for a reversible addition − fragmentation chain-transfer (RAFT) reaction. The resulting polymers had tuneable stiffness and enhanced solubility compared to gelatin. The biohybrid gel also showed good biocompatibility, with MCF-7 cells forming larger spheroids when encapsulated within the biohybrid gel when compared to an unfunctionalized polyethylene-glycol (PEG) gel. Furthermore, due to the ionic crosslinking in the biohybrid gel, spheroids can be retrieved by digesting the matrix using 10 × phosphate-buffered saline (PBS). Retrieved cells were shown to be viable which allows for the potential of downstream analysis. Thus, this study highlights the potential of hybrid gelatin–PEG hydrogels for 3D cell culture.

**Graphical abstract:**

The biohybrid gelatin (Gelatin-SPMA) is crosslinked with a positively charged polymer (PEG-MAETMA) to form a gel within seconds. MCF-7 cells survived encapsulation and formed spheroids over 7 days. 10x phosphate buffered saline (PBS) was then used to digest the hydrogel, allowing for the recovery of encapsulated spheroids.

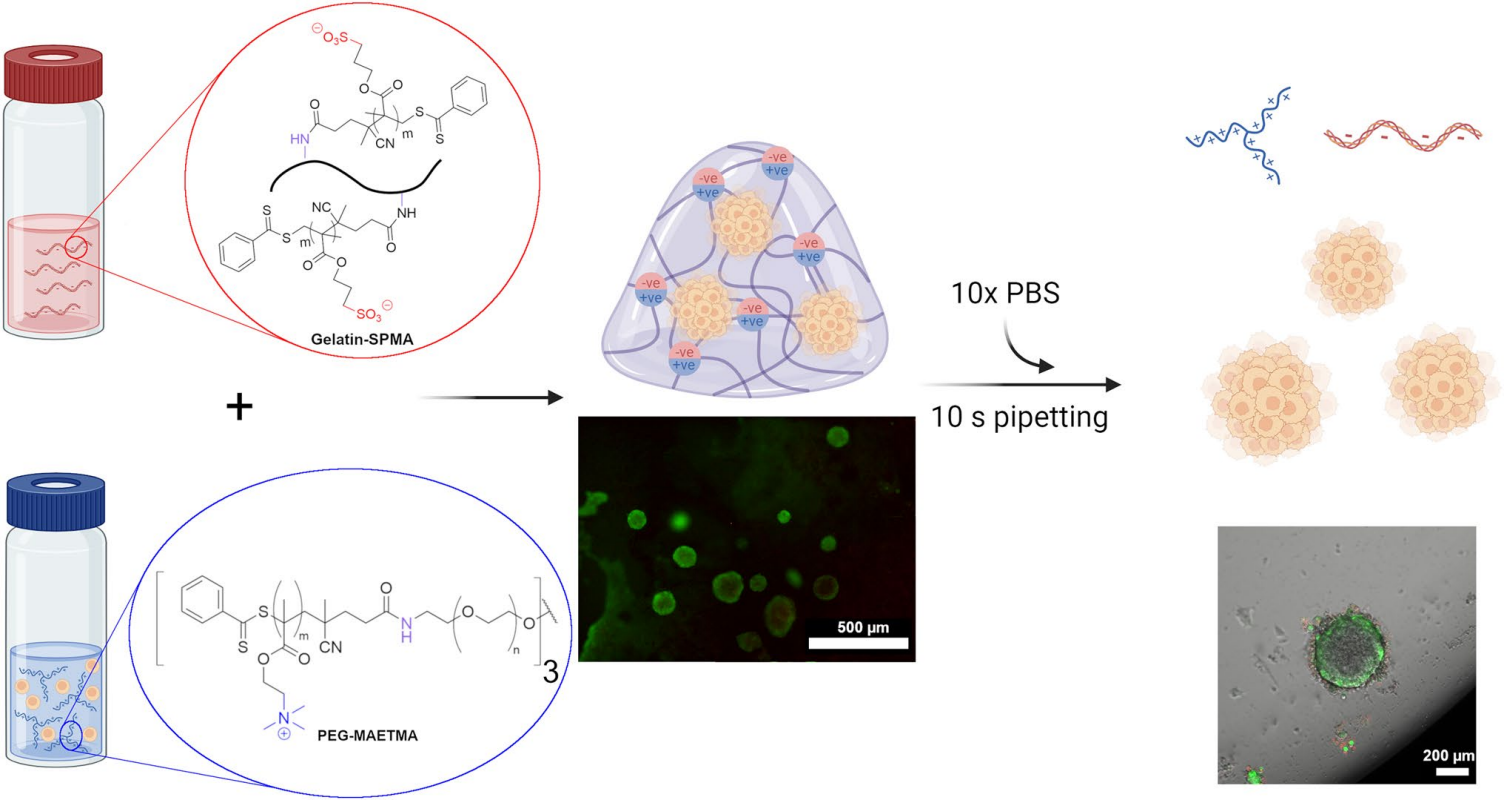

**Supplementary Information:**

The online version contains supplementary material available at 10.1007/s13233-025-00380-z.

## Introduction

With the biomaterials field increasingly adopting three-dimensional (3D) cell culture methods, the development of materials suitable for mimicking the extracellular matrix (ECM) is a highly valuable undertaking. This is largely because 3D in vitro cell models have been shown to reflect a cell’s native microenvironment more accurately than traditional two-dimensional (2D) cell culture [1–3]. Hydrogels formed via the crosslinking of polymers are ideal for the design of ECM mimetics as their porous network and fibrous structure are very similar to the ECM and can support cell adhesion and proliferation [4–6].

Hydrogels can be generally classified as natural or synthetic. Natural polymers such as collagen or gelatin are well established and have been shown in many literature examples to be useful in a wide range of applications. Naturally occurring hydrogels advantageous bioactive properties and the ability to promote cellular functions [7, 8]. In particular, Matrigel, which is derived from Engelbreth–Holm–Swarm mouse sarcomas, is regarded as one of the gold standards in commercially available ECM mimetics. The knowledge obtained from the use of Matrigel has sparked the search for alternatives with greater batch-to-batch control, which has led to synthetic hydrogels such as PEG-based hydrogels and self-assembled peptide hydrogels. Synthetic hydrogels are capable of forming highly defined networks and can be modified with peptide sequences to mimic naturally occurring biological motifs in the ECM [9, 10]. Furthermore, synthetic hydrogels possess a good degree of mechanical tuneability allowing them to mimic the stiffness of a wide range of tissues [11, 12]. There exists an opportunity to combine biological and synthetic polymers to create a superior material that has inherent bioactive cues and good tuneability.

In this paper, we developed a biohybrid gel to combine the versatility of synthetic polymers and the biological relevance of natural polymers. Gelatin is a naturally derived polymer that has been shown in the literature to be easily modifiable due the amino acid residues in its backbone [13, 14]. Gelatin methacryloyl (GelMA) is a classic example of a modified gelatin which is the result of a conjugation reaction of gelatin and methacrylic anhydride [15, 16]. Therefore, gelatin was selected as the candidate for the natural component of a biohybrid gel. The selection of a synthetic component of a biohybrid gel is more versatile, with many choices in the literature. Recently, an ionically crosslinked hydrogel comprised polyelectrolyte-based poly(ethylene) glycol (PEG) was reported by Nguyen et al. and Suwannakot et al. to have a fast gelation, good chemical modifiability, ease of synthesis, and controlled degradation for the recovery of cells [12, 17]. Therefore, we have chosen this polyelectrolyte PEG hydrogel for the synthetic component owing to its versatility. We were able to successfully synthesise the biohybrid ionically crosslinked gelatin hydrogel and show its versatility in physical tuneability, cytocompatibility, and cell recovery. The hydrogels in this work target a lower stiffness which mimics the soft tissues found in human breast tissue. To show the cytocompatibility of this ECM mimic, MCF-7 cells were cultured in the biohybrid hydrogel and measured for viability and spheroid morphology.

## Materials and methods

### Materials

Gelatin sources (cold water fish, porcine skin, and bovine skin), 4-cyano-4-(phenylcarbonothioylthio) pentanoic acid *N*-succinimidyl ester (CPADB > 97%), and 3-sulfopropyl methacrylate potassium salt (SPMA, 98%) were purchased from Sigma-Aldrich. Ninhydrin and glycine were purchased from Ajax Finechem. 4,4′-Azobis (4-cyanovaleric acid) (ACVA, 98%) was purchased from Alfa Aesar. Dialysis tubing (MWCO 10,000 kDa) was from ThermoFisher Scientific. Phosphate-buffered saline (PBS, 0.1 M, pH 7 and pH 10), acetate buffer (0.1 M, pH 5.5), acetone, and ethanol (50% (v/v)) were all prepared with high purity Milli-Q water. Dulbecco’s Modified Eagle Medium (DMEM), foetal bovine serum (FBS), and penicillin–streptomycin (P/S) used in cell culture was purchased from Gibco. AlamarBlue™ and LIVE/DEAD™ kits were purchased from Thermo Fisher Scientific. All solvents used for synthesis and purification were analytical grade. Deuterated solvents, such as acetone-d6 (C_2_D_6_O, 99.9%, 1H = 2.05 ppm) and deuterium oxide (D_2_O, 99.9%, 1H = 4.80 ppm), were purchased from Cambridge Isotope Laboratories, Inc.

### Quantification of primary amine in gelatin

A standard curve was generated using glycine as the source of primary amine. The glycine standard solutions were prepared by diluting a stock solution of glycine (1 mg/mL) in acetate buffer (0.2 M, pH 5.5), resulting in a 7.5, 15, 22.5, 30, and 37 mg/mL glycine standard solutions. Acetate buffer was used as a blank solution. Gelatin solutions were made at 1, 2, and 4 mg/mL concentrations by dissolving gelatin (sourced from cold water fish, porcine skin, and bovine skin) in acetate buffer (0.2 M, pH 5.5) to give the final concentrations of 1, 2, and 4 mg/mL for each source of gelatin. Ninhydrin (8% w/v in acetone, 1 mL) was added to 4 mL of each standard and unknown solutions, followed by constant stirring at 100 °C. The samples were cooled for 5 min in an ice bath. Ethanol (50% v/v in water, 1 mL) was added and each solution was vortexed for 30 s. The absorbance was then measured at 570 nm for the standard solutions and gelatin unknowns. A standard curve was generated and the unknowns were calculated based on the curve.

### Synthesis of 4-cyano-4-(phenylcarbonothioylthio) pentanoic acid N-succinimidyl ester modified gelatin

In a typical synthesis, gelatin type B (1 g, 1.33 × 10^–5^ mol) was dissolved in PBS (pH 10), (100 mL). 4-Cyano-4-(phenylcarbonothioylthio) pentanoic cid *N*-succinimidyl ester (CPADB) (0.3 g, 4.52 × 10^–4^ mol) was added to the solution. The reaction was stirred at 40 °C for 24 h. The reaction was then filtered and washed with water. The filtrate was then dialysed against water (SnakeSkin™ dialysis tubing, 10 kDa molecular weight cut-off) for 2 days and then lyophilised for 3 days to yield gelatin modfiied with CPADB (Gel-CPADB) a pink solid (0.850 g; 50% yield; ^1^H NMR (600 MHz, D2O): δ (ppm) 7.57 (br, 2H,Ar–H), 7.43 (br, 1H, Ar–H), 7.35 (br, 2H, Ar–H), 7.20 (br, 11H, Phe), 4.80 (s, 2H, CH2), 4.49(br, Glu), 4.26 (br, Ala/Val), 3.84 (br, Gly), 3.52 (br, Pro), 3.08 (br, Arg), 2.60 (br, Asp/Met), 2.19 (br, Glu/Pro), 1.91 (br, Val), 1.53 (br, Arg), 1.28 (br, Ala/Ile), 1.12 (br, Thr/Ile) and 0.80 (br, Val/Leu/Ile). IR (ATR): n_max_ 3320 (b), 1636.71 (sh), 1541.58 (sh), 1449.37 (sh), 1399.95 (sh), 1238.69 (sh)).

### Synthesis of 3-sulfopropyl methacrylate modified gelatin

Polymerisation was achieved via the reversible addition − fragmentation chain-transfer (RAFT) reaction. In a typical polymerisation reaction, Gel-CPADB (100 mg, 7.8 × 10^–3^ mol), 3-sulfopropyl methacrylate (SPMA) (964 mg, 3.9 × 10^–3^ mol), and 4,4-Azobis(4-cyanovaleric acid) (ACVA) (0.044 mg, 1.6 × 10^–7^ mol) were dissolved in Milli-Q water (2.6 mL). The reaction vessel was sealed and purged with argon gas for 1 h. The vessel was then stirred at 70 °C for 48 h. Amount of SPMA and stirring time were adjusted to achieve other chain lengths. Polymerisation was terminated by unsealing the reaction and exposing it to air. The crude product was analysed for reaction completeness and then dialysed against Milli-Q water (SnakeSkin™ dialysis tubing, 10 kDa molecular weight cut-off) for 5 days. The solution was then lyophilised for 2 days, giving the 3-sulfopropyl methacrylate modified gelatin (Gelatin–SPMA) as a white, fluffy solid (0.513 g, 70% yield, 1H NMR (600 MHz, D2O): δ (ppm) 7.60 (br, 2H, Ar–H), 7.44 (br, 1H, Ar–H), 7.38 (br, 2H, Ar–H), 4.80 (s, 2H, CH2), 4.53 (br, 9H, Glu), 4.26 (br, Ala/Val), 4.07 (br, OCH2), 3.87 (br, Gly), 3.55 (br, Pro), 3.12 (br, Arg), 2.94 (br, H2C-SO3-), 2.07 (br, R1-CH2-R2O and R-CH3), 1.56 (br, 14H, Arg), 1.30 (br, Ala/Ile), 0.85 (br, R1-CH2-R2).

### Synthesis of [2-(methacryloyloxy)ethyl] trimethylammonium modified PEG

[2-(methacryloyloxy)ethyl] trimethylammonium modified PEG was synthesised according to a previously reported protocol [12, 17]. Briefly, 20 kDa 3-arm PEG-NH_2_ (1.0 g, 6.6 × 10^–5^ mol) and CPADB-NHS (0.090 g, 2.4 × 10^–3^ mol) were dissolved in PBS. The mixture was stirred at room temperature for 24 h. The mixture was dialysed against water with water changes every 30 min for 2 h. The product was then lyophilised to give the RAFT functionalised PEG (PEG-CPADB) as a pink solid. PEG-CPADB (0.1 g, 6.3 × 10^–6^ mol) was dissolved with [2-(methacryloyloxy)ethyl] trimethylammonium (MAETMA) (0.54 g, 2.6 × 10^–3^ mol), and ACVA (0.1 g, 6.3 × 10^–6^ mol) were dissolved in 5 mL of water. The solution was purged with argon for 30 min. The reagents were then heated to 70 °C and stirred overnight. The resulting [2-(methacryloyloxy)ethyl] trimethylammonium modified PEG (PEG-MAETMA) was then dialysed against water with water changes every 30 min for 2 h. Monomer conversion was determined using a method reported in literature and was determined to be 60%, giving 150 repeating units per arm [12, 17]. The product was lyophilised and collected as a white powder (^1^H NMR, 400 MHz, D_2_O: δ (ppm), 4.44 (br, 2H, COO-CH_2_), 3.79 (br, 2H, COO-CH_2_), 3.62 (s, 4H, OCH_2_CH_2_), 3.21 (br, 9H, N(CH_3_)_3_), 1.96 (br, 2H, CH_2_), 1.02 (br, 3H, CH_3_).

### Hydrogel formation

The ionically crosslinked hydrogel was formed by dissolving Gelatin–SPMA in Dulbecco’s modified eagle medium (DMEM) at a concentration of 10% w/v (100 mg in 1 mL). PEG-MAETMA was also dissolved in DMEM at a concentration of 10% w/v (100 mg in 1 mL). The two solutions were then combined in equal volumes to form a gel. Volumes used throughout this work changed depending on the experiment.

### Rheology

Rheology was performed using an Antor Paar MCR302 Modular Compact Rheometer with a PP25 25 mm parallel plate set to a gap height of 1 mm. Hydrogel formation was performed as mentioned above to a final hydrogel volume of 600 mL. The sample was subject to a time sweep, frequency sweep, and strain sweep which were performed as follows:

- Time sweep

Frequency was set to 1 Hz and strain was set to 1% and both were kept constant. Temperature was set to 37 °C. Measurements were taken every 2 s with a total of 450 measurements taken.

b.b.b.b. Frequency sweep

Strain was kept constant at 1%. Frequency was decreased from 10 Hz to 0.1 Hz (logarithmic ramp). Temperature was set to 37 °C. Measurements were recorded every 2 s with a total of 150 measurements taken.

c.c.c.c. Strain sweep

Frequency was kept constant at 1 Hz. Strain was increased from 0.1% to 100% (logarithmic ramp). Temperature was set to 37 °C. Measurements were recorded every 2 s with a total of 150 measurements taken.

### Cell culture

MCF-7 were purchased from ATCC and cultured in DMEM media supplemented with 10% FBS and 1% P/S. Cells were maintained in a humidified atmosphere containing 5% CO_2_ at 37 °C and were tested to be mycoplasma-free. The cell line was authenticated using short tandem repeat profiling at Kinghorn Centre for Clinical Genomics, Australia.

### AlamarBlue assay

AlamarBlue assays were used to measure cell proliferation over 7 days with measurements taken on days 1, 3, and 7. Cells were seeded in 10, 30, and 45 repeating unit (RU) Gelatin–SPMA at a concentration of 5 million cells/mL. Gelatin–SPMA were prepared at a final concentration of 5% w/v and a volume of 5 mL. AlamarBlue was prepared at a concentration of 10% v/v in DMEM. At 16 h prior to each time point, media was aspirated from the desired wells and replaced with the 10% v/v AlamarBlue solution. Fluorescence measurements were made using a Clariostar plate reader at an excitation wavelength of 560 nm and an emission wavelength of 590 nm.

### Cell retrieval

Cells were retrieved from the Gelatin–SPMA hydrogels at day 7. After media was aspirated from the desired wells, 100 mL of 10 × DPBS was added to each well with gentle pipetting for 10 s. The dissociated gels in 10 × DPBS was then immediately deposited into a vial containing 1 mL of DMEM to reduce osmotic stress.

### LIVE/DEAD assay

Cell viability analysis was performed using a LIVE/DEAD kit for mammalian cells from Invitrogen. Cells were seeded in 10, 30, and 45 RU Gelatin–SPMA at a concentration of 5 million cells/mL for a period of 7 days. At day 7, the media was aspirated from the wells. The cells were then stained with 200 mL of LIVE/DEAD solution [10 μM ethidium homodimer-1 (EthD1) and 5 μM calcein AM in DMEM]. Cells were incubated for 30 min and then imaged at 5 × magnification using a CellDiscoverer 7 microscope.

## Results and discussion

### Synthesis of gelatin–PEG hybrid

**Scheme 1:**
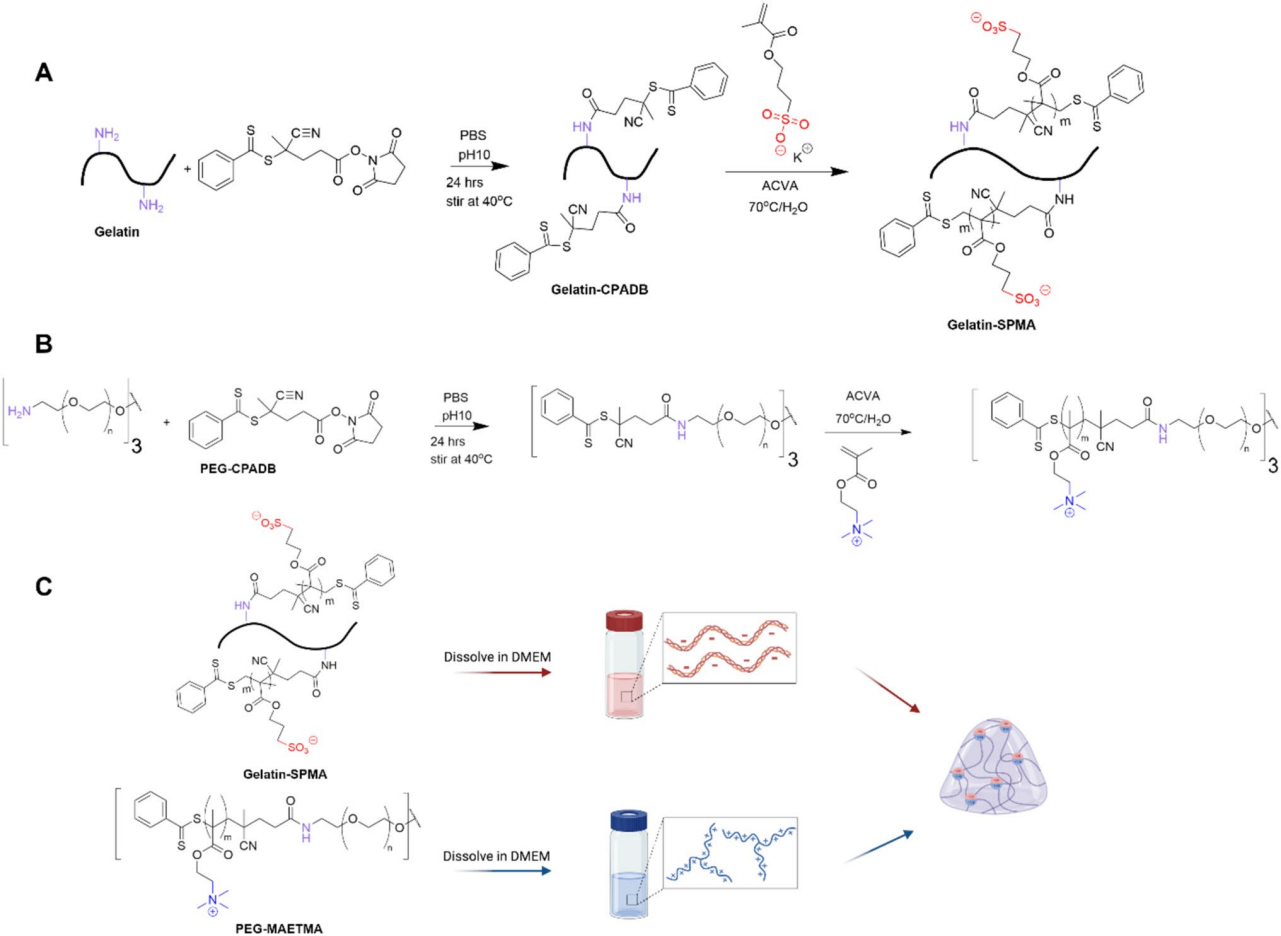
Schematic of the synthesis of gels. **A** Synthetic strategy of functionalising Gelatin. Gelatin was first conjugated with RAFT agent CPADB via aminolysis. Reaction was performed in PBS at pH 10 and stirred for 24 h at 40 °C to yield Gel-CPADB. RAFT polymerisation was then performed with Gel-CPADB and SPMA to form the negatively charged gelatin polymer to form Gelatin–SPMA. **B** Synthetic strategy of PEG-MAETMA as reported in the literature. **C** General gelation protocol of Gelatin–SPMA crosslinked with PEG-MAETMA

The gelatin-based hydrogel used in this study was formed by ionically crosslinking two oppositely charged polymers. The positively charged polyethylene glycol (PEG) with [2-(methacryloyloxy)ethyl] trimethylammonium (MAETMA) polymer was synthesised using a modified version of a previously reported protocol [12, 17]. The negatively charged modified gelatin was synthesised via a two-step process (Scheme 1A). The first step in the synthesis was an aminolysis reaction between primary amines along the backbone of the gelatin with the carbonyl group of the reversible addition − fragmentation chain-transfer (RAFT) agent. To determine the amount of RAFT agent needed, the approximate concentration of primary amine in gelatin derived from different sources (derived from fish, porcine, and bovine) was quantified using a ninhydrin assay. A calibration curve was generated using the absorbance of the primary amines in standard solutions containing different concentrations of glycine (Fig. S1). Gelatin samples from fish, porcine, and bovine were then fitted to the curve which revealed amine concentrations of 1.63 × 10^–4^, 3.33 × 10^–4^, and 3.69 × 10^–4^ mol/g, respectively. This revealed the bovine derived gelatin to have the highest concentration of primary amines and was chosen as the ideal candidate for further modification. The CPADB conjugation was performed in basic conditions and was confirmed using ^1^H NMR (Fig. S2) by the peak at 7.5 ppm which corresponds to the phenyl group in the CPADB. The second step was the well-documented RAFT polymerisation of the SPMA monomers to the CPADB modified gelatin (Gelatin-CPADB). The length of the polymer chain was controlled by the molar equivalent of SPMA monomer units and reaction time. The chain length was confirmed by ^1^H NMR (Fig. S3) using the peak at 2.9 ppm which corresponds to the methylene group next to the sulphate end group on the SPMA [17]. This peak was compared to the aromatic peaks at 7.5 ppm to determine the number of protons associated with the methylene group. This confirmed the synthesis of chains of 15, 30, and 45 repeating units of SPMA along the gelatin backbone.

**Fig. 1:**
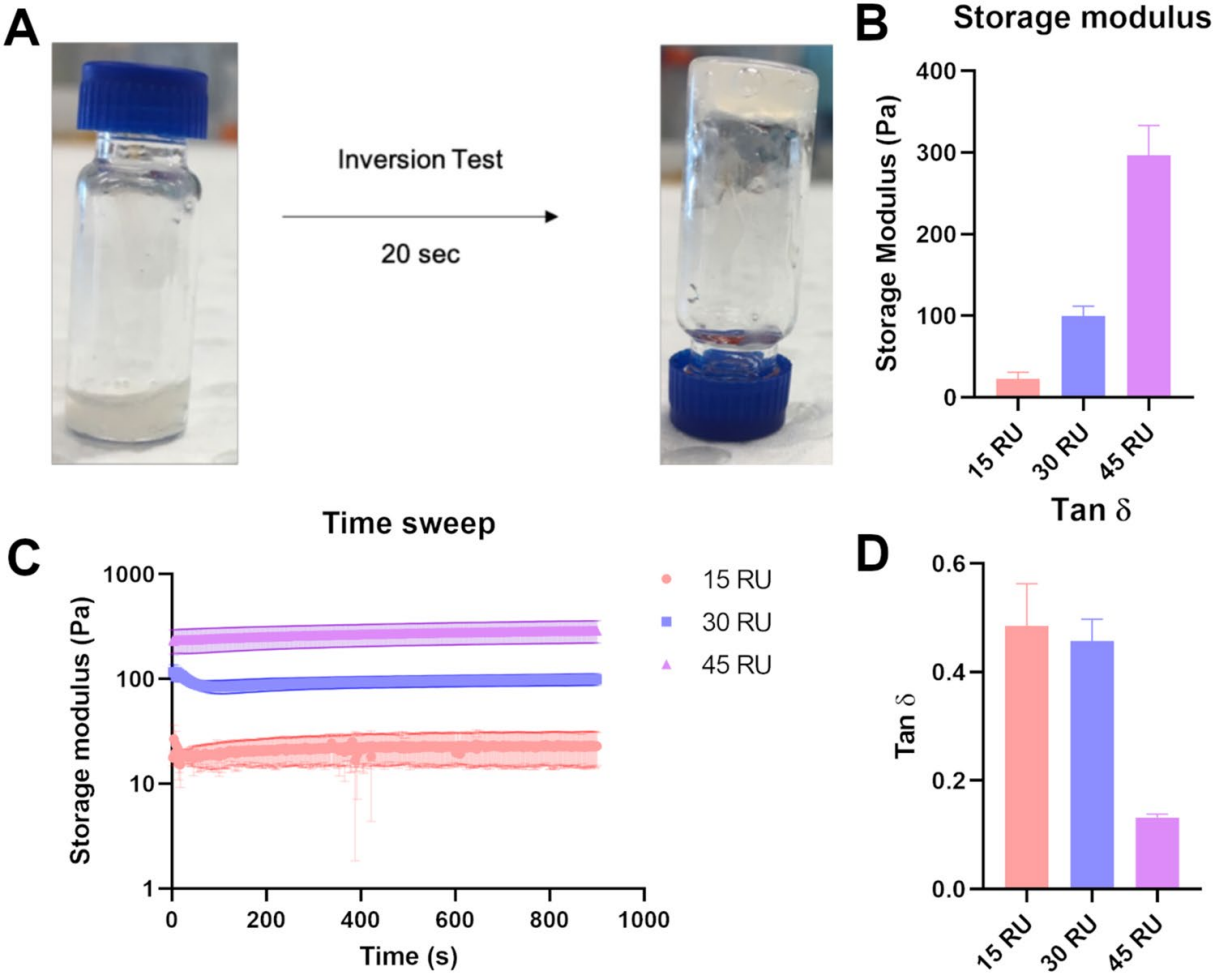
Gelation properties of the Gelatin–SPMA (15, 30, and 45 RU) crosslinked with PEG-MAETMA with **A** a representative inversion test image taken 20 s after mixing. Rheological data showing **B** storage modulus (*G*’), **C** time sweeps, and **D** tan of Gelatin–SPMA 15, Gelatin–SPMA 30, and Gelatin–SPMA 45

Hydrogels were formed by crosslinking Gelatin–SPMA with PEG-MAETMA which were prepared as separate solutions at initial concentrations of 10% w/v. The oppositely charged polyelectrolytes are stabilised via electrostatic interactions to give a gel (Scheme 1). Testing revealed that the gelation occurred within 20 s which is common for gels that form using electrostatic interactions (Fig. 1A and Fig. S4) [18–20]. It should be noted that increasing the chain length of the arms decreases overall solubility which is a trend reflected in literature [21–23]. Going beyond 45 RU results in low solubility in DMEM and causes difficulties in handling. Therefore, in testing mechanical characterisation and cytocompatibility assessments, arm length was limited to 45 RU. Storage modulus (*G*’) was used as the indication for stiffness. As seen in Fig. 1C, the storage modulus increases with increasing arm lengths. This can be attributed to the presence of more crosslinking sites due to the larger amount of charges on the gelatin arms, giving storage modulus values of 23, 97, and 281 Pa for 15, 30, and 45 RU, respectively (Fig. 1B). These values can be converted to Young’s modulus values by applying a previously reported formula which resulted in Young’s modulus values of 67, 281, and 815 kPa, respectively (Equation S1) [24]. These values are within the range of reported literature Young’s modulus for breast tissue [25, 26]. This trend is reflected in the literature in which more charged repeating units increases the stiffness of the gel [12, 17]. The tan δ values (a ratio of loss modulus to storage modulus) are below 1 for all gels which further indicates that the materials are exhibiting gel characteristics (Fig. 1D). Notably, the tan δ value for the 45 RU is significantly lower than 15 RU and 30 RU Gelatin–SPMA. Interestingly, Gelatin-CPADB with no polyelectrolyte arms did not form a gel when mixed with the PEG-MAETMA crosslinker (Fig. S4). Furthermore, rheology measurements were performed at 37 °C and demonstrates that the biohybrid Gelatin–SPMA remains thermally stable at 37 °C. Unfunctionalised gelatin is known to be thermally unstable and does not remain as a gel at 37 °C [27, 28]. This suggests that the ionic crosslinking provided by the polyelectrolyte arms is the driving force for the gelation process and allows the gelatin component to remain stable at physiologically relevant temperatures. Interestingly, the Gelatin–SPMA solution did not form a gel at 10% w/v despite gelatin normally forming a gel at this concentration and only formed a gel when crosslinked with the positively charged polymer. This is likely due to the basic pHs used in the conjugation reaction of CPADB. As gelatin is known to fragment in alkaline conditions, it is hypothesised that the gelatin backbone is fragmented into smaller, more soluble pieces [29, 30].

**Fig. 2:**
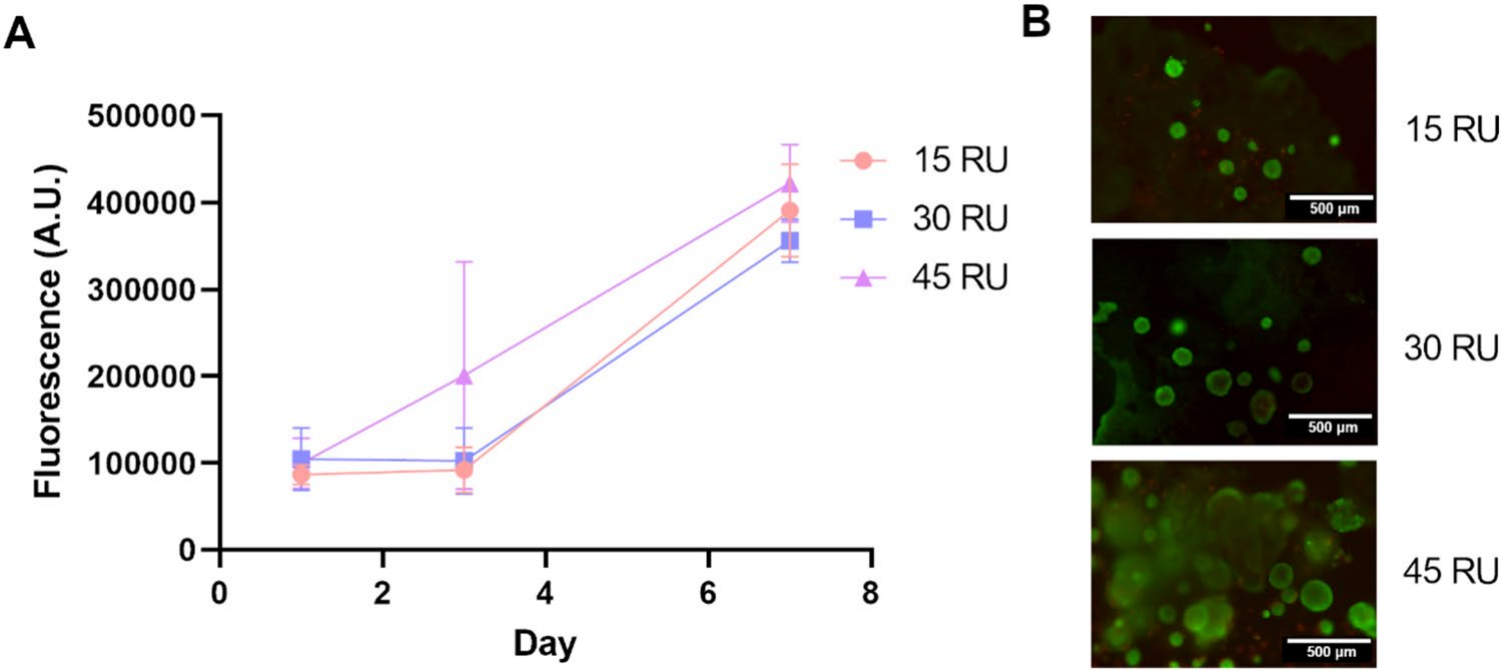
Cell viability and proliferation of MCF7 in gels as determined by AlamarBlue and LIVE/DEAD assays. **A** Cells were incubated over a period of 7 days. Proliferation was measured using AlamarBlue at days 1, 3, and 7. Data show a gradual increase in fluorescence as determined by the colour change in AlamarBlue. **B** At day 7, cells were confirmed to be alive as seen by the predominantly green colour of the LIVE/DEAD stain. Scale bars are 500 μm

Next, the cytocompatibility of the Gelatin–SPMA was evaluated to assess its capability as an extracellular matrix mimic. MCF-7 cells were seeded in the gels at an initial concentration of 5 million cells/mL and then monitored over 7 days with AlamarBlue and LIVE/DEAD. Measurements for AlamarBlue were taken at days 1, 3, and 7. As seen in Fig. 2A, the MCF-7 cells proliferated over the 7 days to a fourfold increase in proliferation. After 7 days, there was no significant difference between each of the conditions. The LIVE/DEAD assay was utilised to visually assess the viability of the MCF-7 cells after the 7 days. Cells were stained with calcein AM, for live cells (green), and ethidium homodimer-1, for dead cells (red). As can be seen in Fig. 2B, MCF-7 cells were observed to be predominantly alive as indicated by the green cells, while dead cells were minimally observed. These results correlate well with the findings of the AlamarBlue assay. These consistent results across both assays confirms that the Gelatin–SPMA hydrogels is capable of supporting the growth and proliferation of cells.

**Fig. 3:**
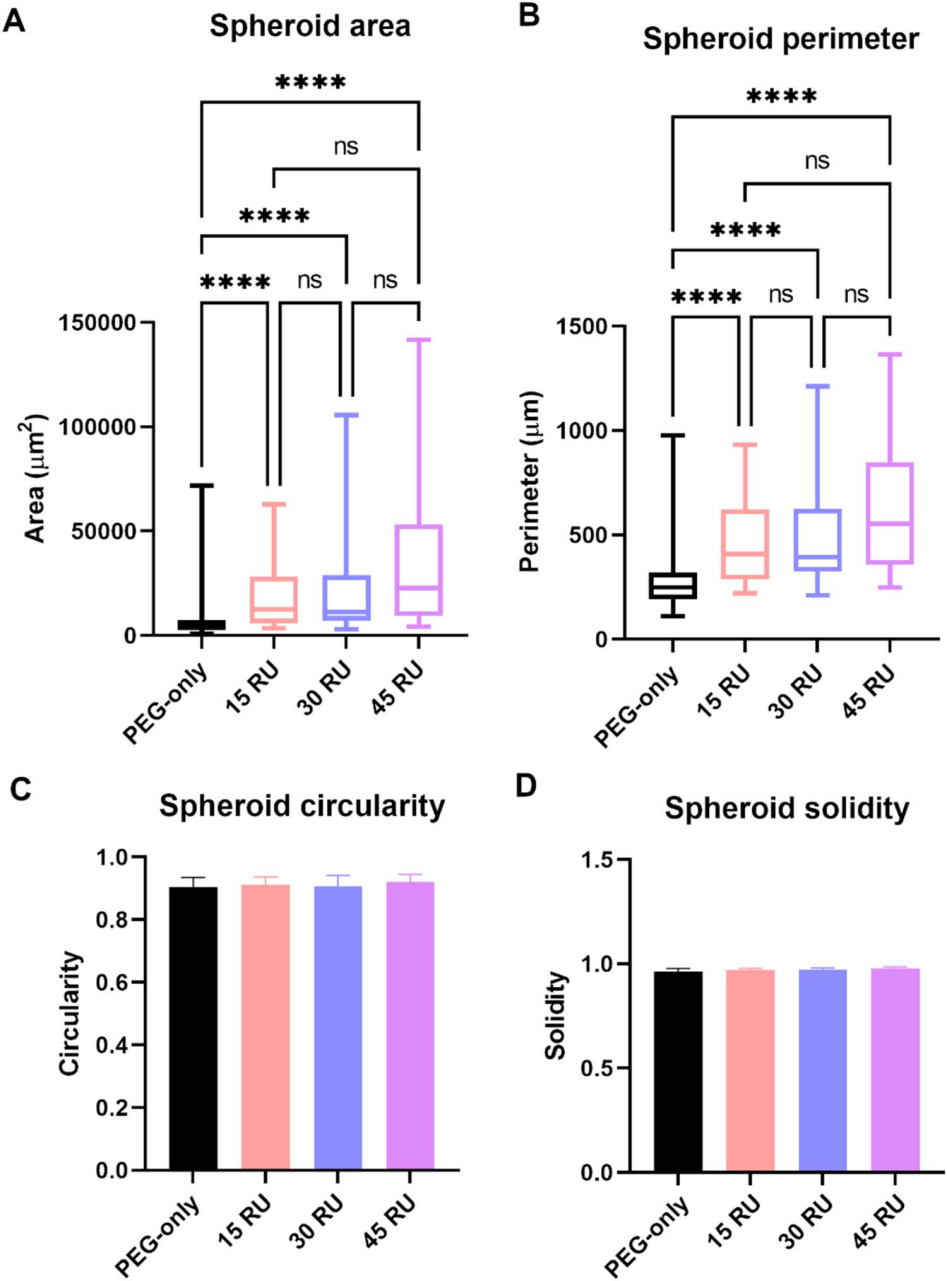
Spheroid characteristics of MCF-7 cells after 7 days of culture. Size was measured by **A** area and **B** perimeter. In the PEG-only gel (no gelatin conjugation), the spheroids were significantly smaller than in conditions utilising the gelatin backbone. Morphology was also measured by **C** circularity and **D** solidity. No changes were seen in morphology across all gel conditions. ****: P < 0.0001 as determined by Kruskal–Wallis test

In addition to viability, the morphology of spheroids formed by the MCF-7 breast cancer cells over 7 days was measured for size and shape. This was performed to assess if the fragmentation of the gelatin backbone impacted its ability to support cell growth. Spheroids formed in the 15 RU, 30 RU, and 45 RU Gelatin–SPMA were compared to a PEG-based polyelectrolyte gel without gelatin. The PEG control was synthesised via an RAFT reaction using 3-sulfopropyl methacrylate (SPMA) monomer units to a length of 150 repeating units. Over the 7 days, spheroids formed in the biohybrid Gelatin–SPMA gels were significantly larger than in the PEG condition (Fig. 3A, B). The median value for the perimeter of the spheroids was 12.5 × 10^3^, 11.2 × 10^3^, and 22.9 × 10^3^ μm^2^ in 15RU, 30RU, and 45 RU gels respectively. This is in comparison to 4.5 × 10^3^ μm^2^ in the hydrogel with no gelatin. Similarly, the perimeter was also larger with the median values being 409.2, 392.6, and 554.1 mm for 15 RU, 30 RU, and 45 RU Gelatin–SPMA, respectively, while in the PEG-only sample, the perimeter was 249.5 μm. The increased size suggests that the conjugation of gelatin provides an environment which promotes the formation of spheroids via cell–cell interactions. The largest spheroids were observed in the 45 RU Gelatin–SPMA matrix. This was likely due to this condition having the closest stiffness value to breast tissue. Despite all biohybrid gelatin conditions exhibiting larger spheroids than the PEG-only condition, it should be noted that the spheroid sizes in 15, 30, and 45 RU Gelatin–SPMA were not statistically significantly different from each other. This suggests that at these stiffnesses, the MCF-7 cells are likely unable to distinguish differences in mechanical cues. This is hypothesised to be due to the relatively low stiffnesses of the biohybrid hydrogels as tumour cells tend to form fewer adhesions and respond less to low stiffness environments [31, 32]. The morphology was measure by circularity and solidity of the spheroids which remained the same across all conditions (Fig. 3C, D). This indicates that the Gelatin–SPMA hydrogels not only support larger spheroid formation but also preserve the morphology of the spheroids.

**Fig. 4:**
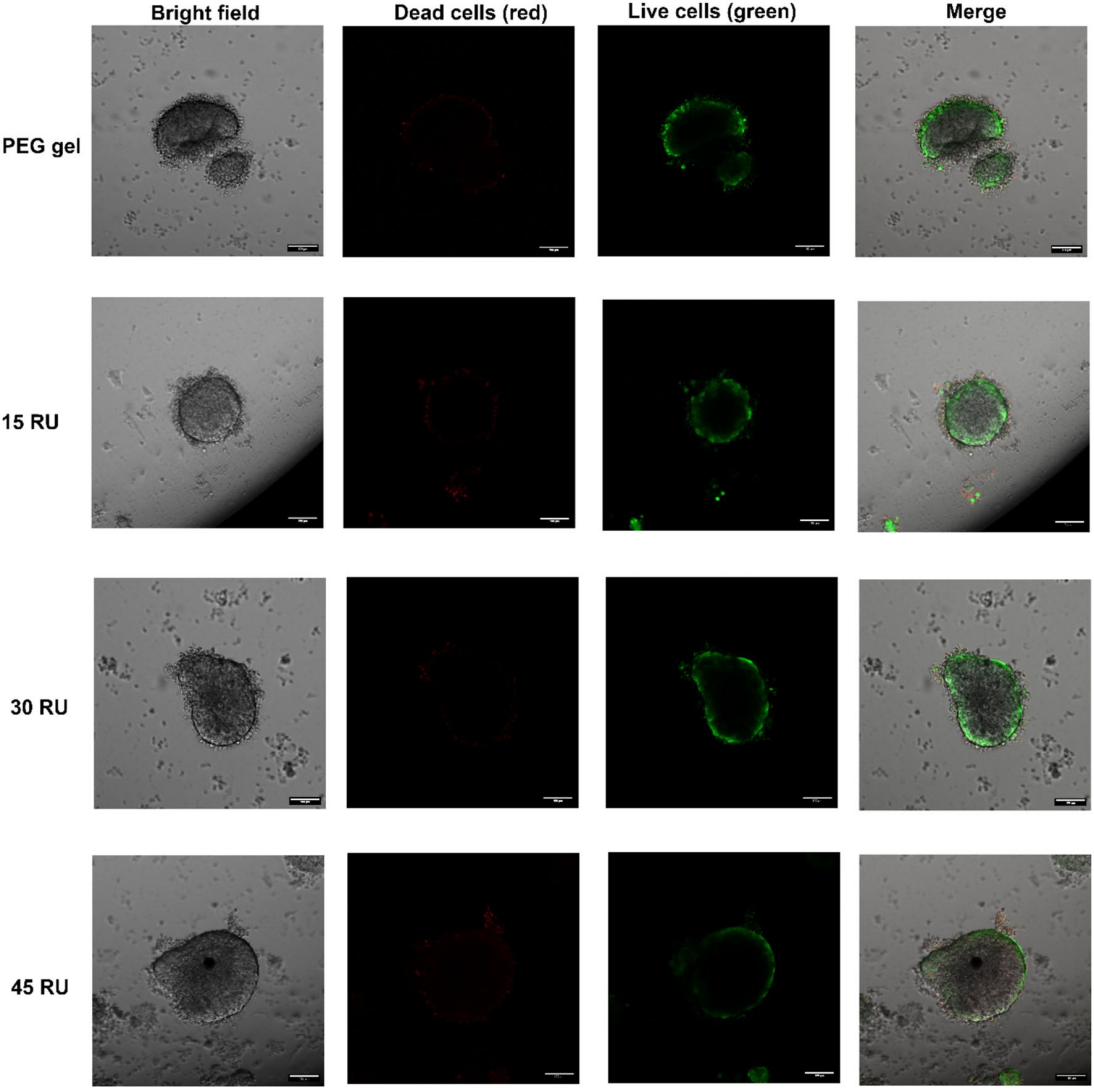
Cell retrieval and LIVE/DEAD of retrieved spheroids in an ionically crosslinked PEG hydrogel, 15 RU Gelatin–SPMA, 30 RU Gelatin–SPMA, and 45 RU Gelatin–SPMA. Representative images are shown as bright field, dead cells in red (ethidium homodimer-1), live cells in green (calcein), and with all channels merged. Scales bars are 100 μm

As the Gelatin–SPMA hydrogel is formed via ionic interactions, the hydrogel is able to be degraded via the addition of excess salt. This allows for the retrieval of released cells which can then be used for downstream analysis. To demonstrate this property, MCF-7 cells were cultured in the Gelatin–SPMA hydrogels and then recovered after 7 days (Fig. 4). Viability of the recovered spheroids were also assessed using LIVE/DEAD. This concept has been reported in the literature with a similar ionically crosslinked gel which was degraded using sodium chloride [12]. In this work, 10 × PBS was used instead of sodium chloride as it was hypothesised to be less harsh on the released cells. Gelatin–SPMA gels were exposed to 10 × PBS solution with 10 s of pipetting to degrade the gels. As shown in Fig. 4, spheroids were successfully released from the gels. LIVE/DEAD was performed on the released cells to assess viability after release. It should be noted that single cells which were released were predominantly dead. This was likely to be due to osmotic shock owing to the 10 × PBS solution. However, spheroids that were released from the matrix were shown to be alive as indicated by the predominant presence of green cells.

## Conclusions

The work presented here shows an elegant and simple combination of two existing concepts: the modification of gelatin, and the RAFT polymerisation reaction. In optimising the synthesis of the Gelatin–SPMA gels, we have succeeded in creating a biohybrid gel with tuneable properties and with enhanced stability at physiologically relevant temperatures. We have shown that the stiffness can be tuned by adjusting the arm lengths [26]. The hydrogels explored in this work are soft, encompassing soft tissues such as breast tissues. We showed that the gel stiffness can be further enhanced by increasing arm length, making it possible to mimic stiffer tissues. This property is valuable as it broadens the potential application of this gel, including stiffer tissues, such as endothelial tissue and fibroblasts. In addition, the ability to achieve these results with minimal changes in materials is extremely important in research, as it reduces the number of variables, allowing for more accurate and consistent comparison across studies. AlamarBlue assays showed that MCF-7 cells cultured in the Gelatin–SPMA gels are able to proliferate over 7 days. The results were confirmed by LIVE/DEAD assays which showed that the cells were alive after 7 days and had formed spheroids. Furthermore, the biohybrid Gelatin–SPMA hydrogels retained the biological function of gelatin despite fragmentation. This was shown in the cell studies as MCF-7 cells formed larger spheroids in the Gelatin–SPMA conditions than in PEG-only gels. The MCF-7 spheroids were also recoverable via application of 10 × PBS while retaining overall cell viability. Thus, this study demonstrates the potential of hybrid gelatin–PEG hydrogels for cell applications with higher ECM replication compared to pure synthetic PEG hydrogels. This property is critical for capturing true cellular behaviour for a better understanding of full biological complexity to drive advancements in both therapeutic development and basic foundational science.

## Supplementary Information

Below is the link to the electronic supplementary material.

Supplementary file1 (DOCX 822 KB)
