# Supplementary material for: Ionically crosslinked biohybrid gelatin-based hydrogels for 3D cell culture
Source: Macromol Res. 2025 Feb 19;33(7):921–31. doi: 10.1007/s13233-025-00380-z (PMC12267368; doi:10.1007/s13233-025-00380-z)
Supplement: Supplementary file 1 — Supplementary file1 (DOCX 822 KB) [file 13233_2025_380_MOESM1_ESM.docx]

**Supplementary Information**

**Eric Y. Du^a, b^, H.T. Kim Duong^a^, M.A. Kristine Tolentino^a, b^, Jacinta L. Houng^a, b^, Panthipa S Eric Y. Du^a,b^, H.T. Kim Duong^a,b^, M.A. Kristine Tolentino^a, b^, Jacinta L. Houng^a, b^, Panthipa Suwannakot^a,b^, Kristel C. Tjandra^a^, Duyen H.T. Nguyen^a,b^, Richard D. Tilley^a, c^, J. Justin Gooding^a, b^***

^a^ School of Chemistry, The University of New South Wales, 2032

^b^ Australian Centre for Nanomedicine, The University of New South Wales, 2032

^c^ Electron Microscopy Unit, Mark Wainwright Analytical Centre, The University of New South Wales, 2032

* Corresponding author email address: [justin.gooding@unsw.edu.au](mailto:justin.gooding@unsw.edu.au)

**
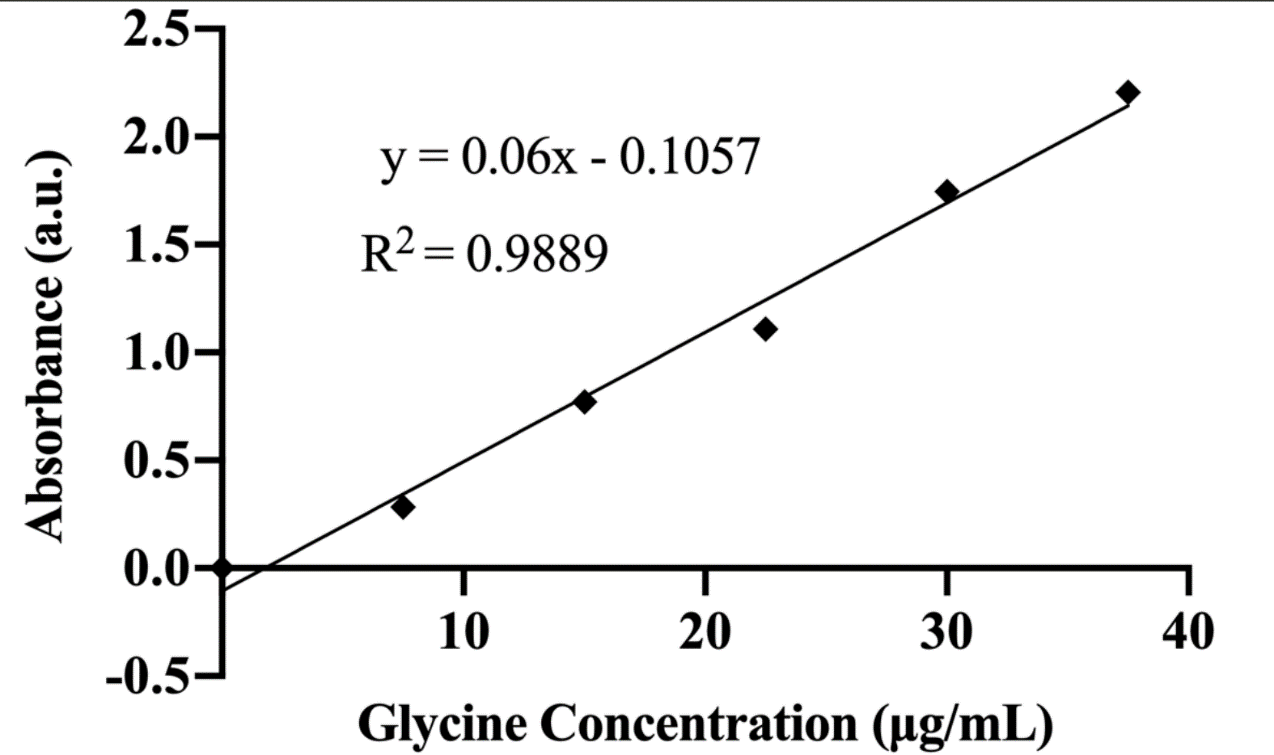
Figure S1.** The calibration curve generated from glycine standard solution of different concentrations.


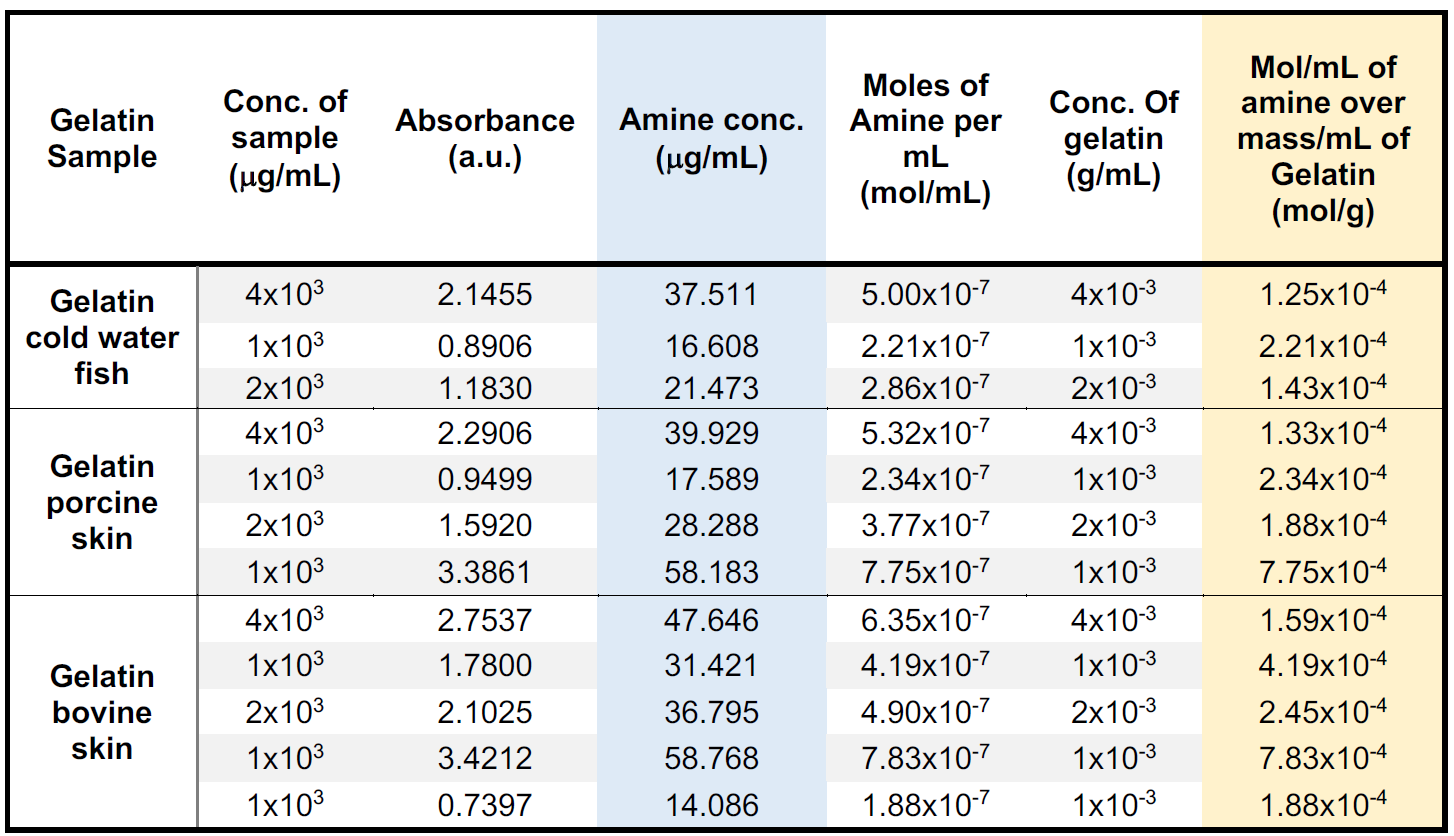
**Table S1.** Concentration of primary amine (mol/g) found in Gelatin Type B from different sources as determined using the calibration curve generated with glycine.

**Table S2.**  Summary of CPADB conjugation reactions with gelatin type B (bovine skin)


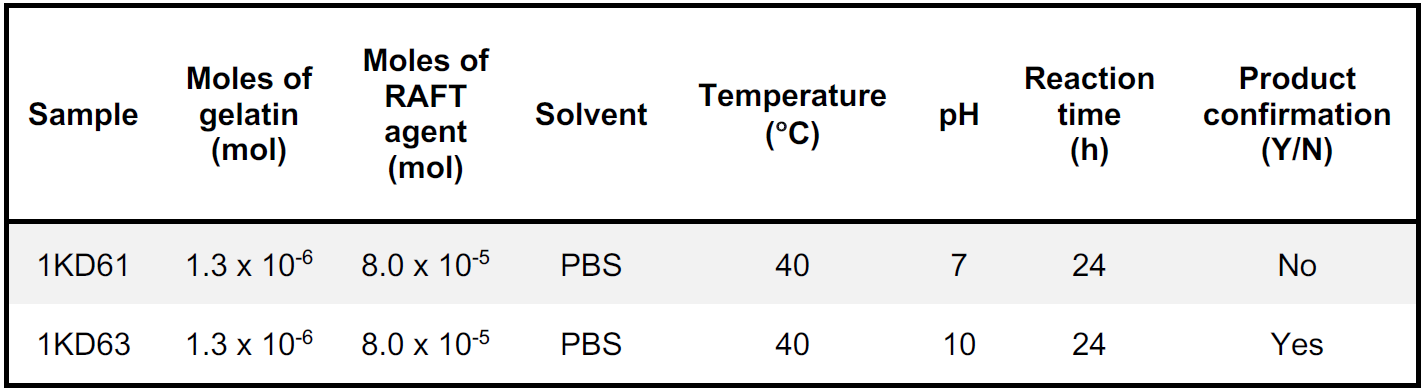


**
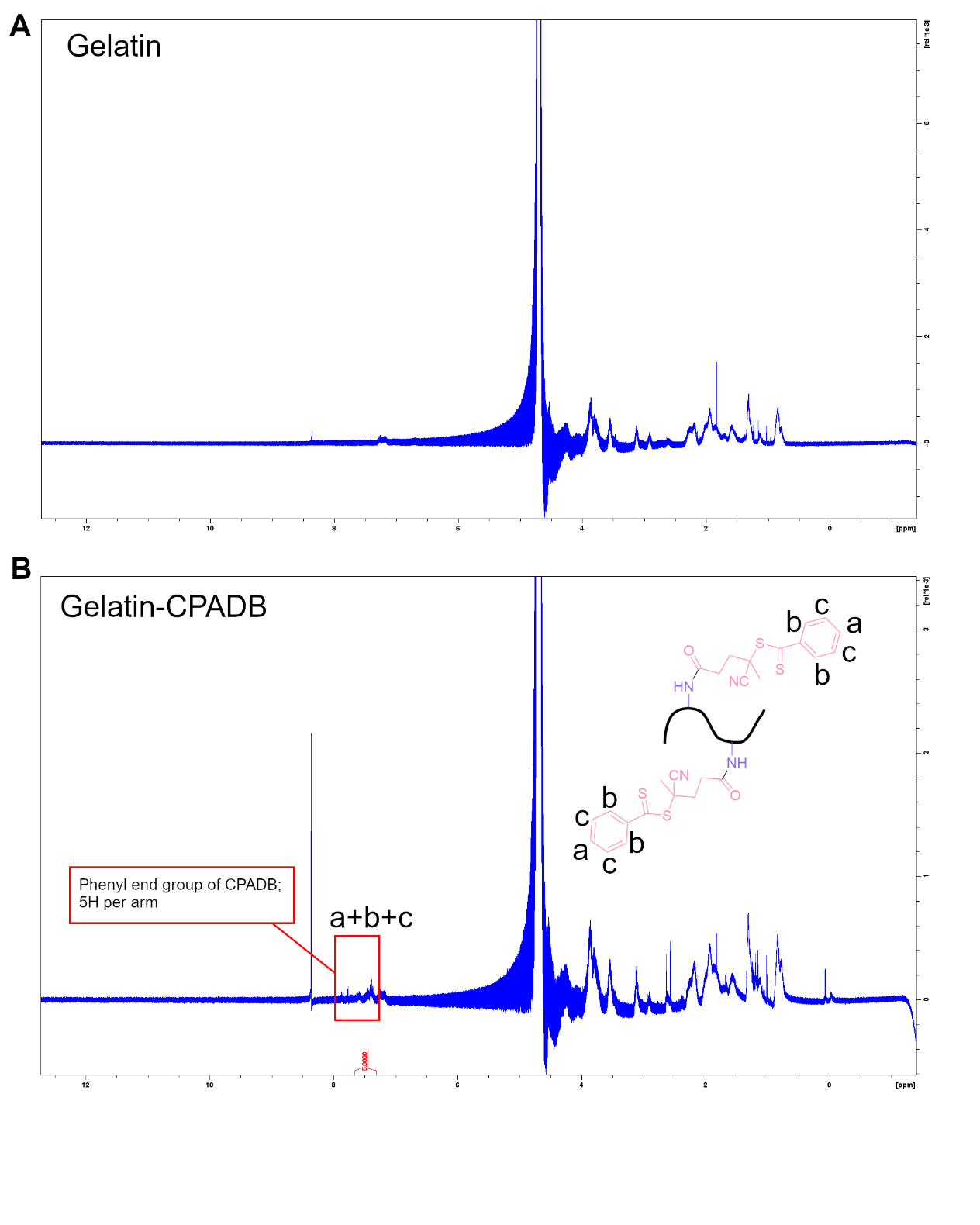
Figure S2.** Stacked ^1^H NMR (400 MHz, D_2_O) of the CPADB conjugation reaction to gelatin performed at **(A)** pH 7 and **(B)** pH 10. The new peak at 7.6 (highlighted yellow) indicates the reaction was successful at pH 10


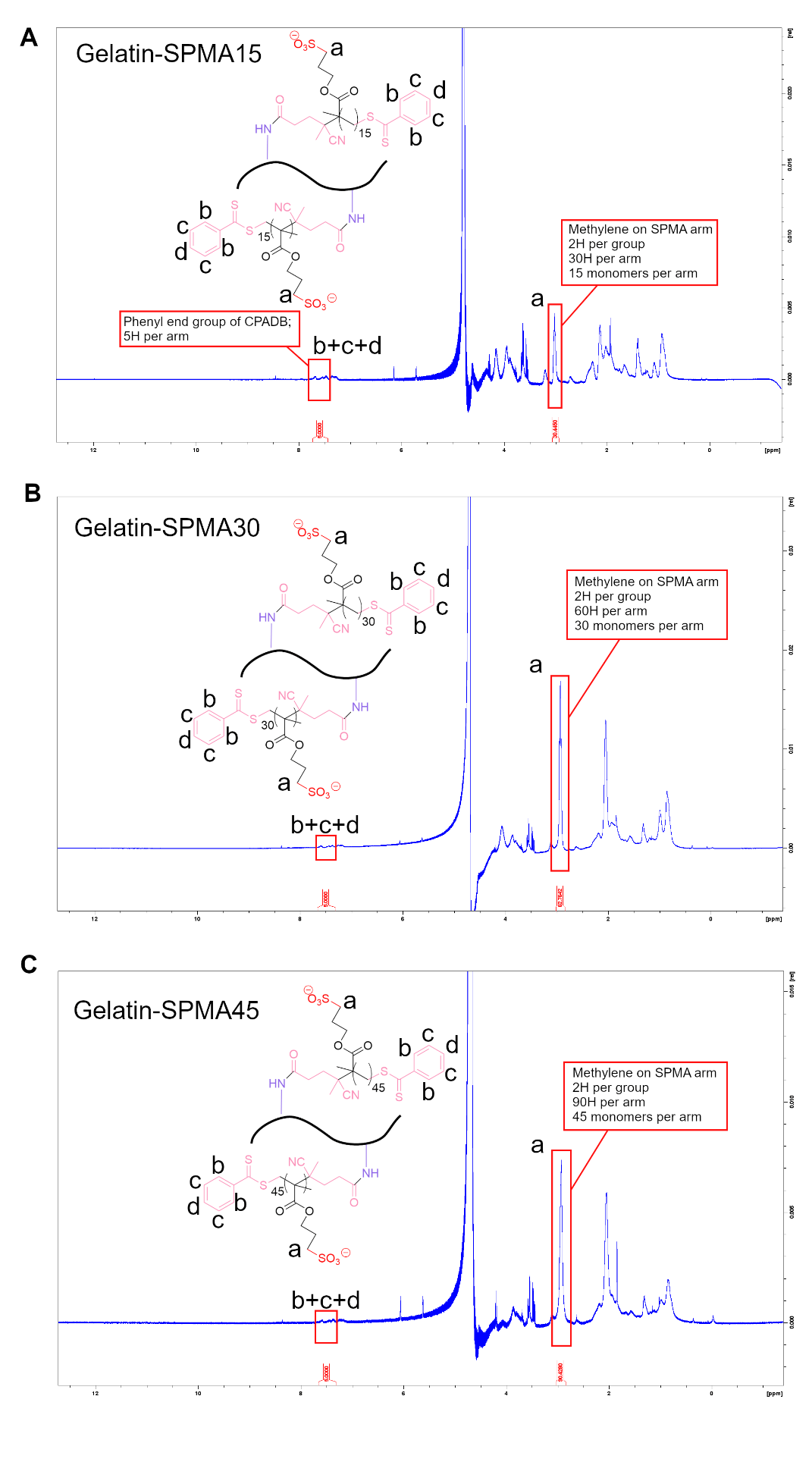
**Figure S3.** Stacked ^1^H NMR (400 MHz, D_2_O) of the **A)** Gel-SPMA15, **B)** Gel-SPMA30, and **C)** Gel-SPMA45. The methylene (CH_2_) peak at 2.9 corresponds to the CH_2_ next to the terminal sulphate group. This peak was used to determine the chain length.


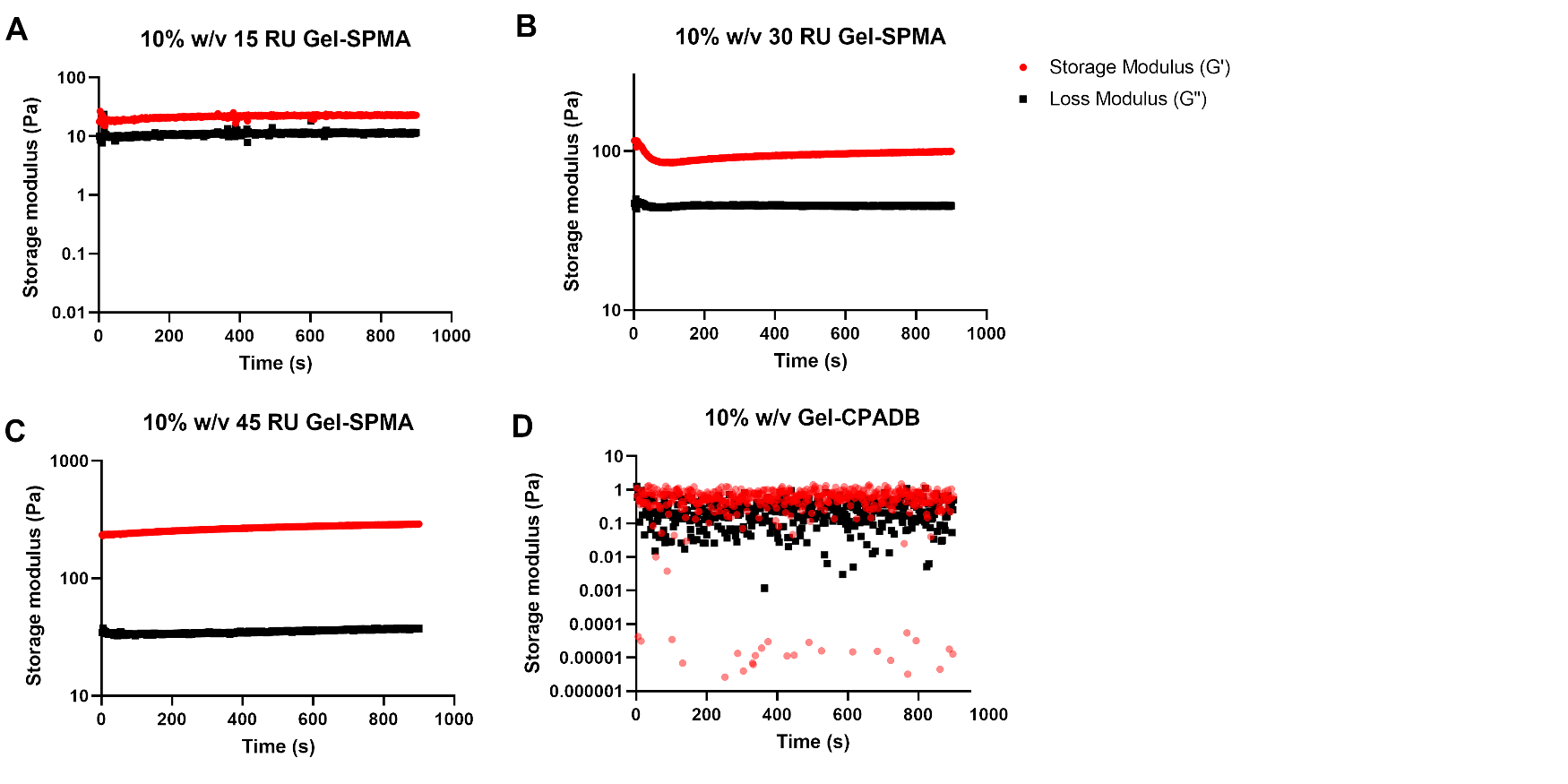
**Figure S4.** Time sweeps tested for 15 minutes for **A)** Gel-SPMA15, **B)** Gel-SPMA30, **C)** Gel-SPMA45, and **D)** GelCPADB. All polymers were prepared at 10% w/v and crosslinked with 10% w/v PEG-MAETMA.

$$E=2G"(1+v)$$

**Equation S1.** Formula for calculating Young’s modulus (E) from shear modulus (G”) where v is the Poisson’s ratio (assumed to be 0.5 for gelatin). Data was obtained in oscillatory rheology using a frequency of 0.1 Hz.
